# Susceptibility of juvenile and adult blood–brain barrier to endothelin-1: regulation of P-glycoprotein and breast cancer resistance protein expression and transport activity

**DOI:** 10.1186/1742-2094-9-273

**Published:** 2012-12-19

**Authors:** Rania Harati, Anne-Sophie Villégier, William A Banks, Aloise Mabondzo

**Affiliations:** 1 CEA, Direction des Sciences du Vivant, iBiTec-S, Service de Pharmacologie et d’Immunoanalyse, Gif-sur-Yvette, F91191, France; 2 Department of Experimental Toxicology, Institut National de l'Environnement Industriel et des Risques (INERIS), BP.2, Verneuil-en-Halatte, 60550, France; 3 GRECC, Veterans Affairs Puget Sound Health Care System, Division of Gerontology and Geriatric Medicine, Department of Medicine, University of Washington, Seattle, WA, USA

**Keywords:** Juvenile and adult blood–brain barrier, Neuroinflammation, Endothelin-1, Brain-to-blood transport, Drug transport, P-glycoprotein, Breast cancer resistance protein

## Abstract

**Background:**

P-glycoprotein (P-gp) and breast cancer resistance protein (BCRP) play a critical role in keeping neurotoxic substances from entering the brain. We and others have previously reported an impact of inflammation on the regulation of adult blood–brain barrier (BBB) efflux transporters. However, studies in children have not been done. From the pediatric clinical perspective, it is important to understand how the central nervous system (CNS) and BBB drug efflux transporters differ in childhood from those of adults under normal and inflammatory conditions. Therefore, we examined and compared the regulation of P-gp and BCRP expression and transport activity in young and adult BBB and investigated the molecular mechanisms underlying inflammatory responses.

**Methods:**

Rats at postnatal day (P) P21 and P84, corresponding to the juvenile and adult stages of human brain maturation, respectively, were treated with endothelin-1 (ET-1) given by the intracerebroventricular (icv) route. Twenty-four hours later, we measured P-gp and BCRP protein expression in isolated brain capillary by immunoblotting as well as by transport activity *in vivo* by measuring the unbound drug partitioning coefficient of the brain (K_p,uu,brain_) of known efflux transporter substrates administered intravenously. Glial activation was measured by immunohistochemistry. The release of cytokines/chemokines (interleukins-1α, 1-β (IL-1β), -6 (IL-6), -10 (IL-10), monocyte chemoattractant protein (MCP-1/CCL2), fractalkine and tissue inhibitor of metalloproteinases-1 (TIMP-1)) were simultaneously measured in brain and serum samples using the Agilent Technology cytokine microarray.

**Results:**

We found that juvenile and adult BBBs exhibited similar P-gp and BCRP transport activities in the normal physiological conditions. However, long-term exposure of the juvenile brain to low-dose of ET-1 did not change BBB P-gp transport activity but tended to decrease BCRP transport activity in the juvenile brain, while a significant increase of the activity of both transporters was evidenced at the BBB in the adult brain. Moreover, juvenile and adult brain showed differences in their expression profiles of cytokines and chemokines mediated by ET-1.

**Conclusions:**

BBB transporter activity during neuroinflammation differs between the juvenile and adult brains. These findings emphasize the importance of considering differential P-gp and BCRP transport regulation mechanisms between adult and juvenile BBB in the context of neuroinflammation.

## Background

The blood–brain barrier (BBB) maintains brain homeostasis and limits the entry of toxins and pathogens into the brain. Adenosine triphosphate-binding cassette (ABC) transporters play a critical role in keeping neurotoxic substances from entering the brain and in transporting toxic metabolites out of the brain [1,2]. These transporters are largely responsible for the multidrug resistance (MDR) phenomenon, which plays a crucial role in treatment failure for several brain diseases such as seizure [3] and human immunodeficiency (HIV-1) infection disease [4,5]. In response to injury or brain diseases [6], the central nervous system (CNS) exhibits inflammatory features, which have effects on the expression and function of BBB efflux transporters in adults [7-9]. Because intricate developmental processes are taking place during the prenatal and postnatal periods, we hypothesized that BBB efflux transporters might also undergo important changes during brain maturation, and might possibly have age-related differences in the inflammatory response. There is now a wealth of evidence that age could have a significant effect on response to cytokines, which, in turn, could modulate BBB efflux transporters expression and activity [10-13] in an age-dependent manner.

Endothelin-1 (ET-1), an arterial vasoconstrictive and vasodilator peptide cytokine [14], is released in several CNS disorders [15-25]. Members of the endothelin family are released by various cell types in brain, including endothelial cells and some glial cells, especially during inflammation [26-29]. In effect, ET-1 is known to be a component of the brain’s innate immune response. It is released during the activation of the brain’s innate immune response [19-31] triggered by a variety of stimuli, including infection, trauma, disease, and cell stress and characterized mainly by glial activation and the release of proinflammatory cytokines and chemokines [32-36]. Previous works in our laboratory as well as studies from other laboratories have shown that proinflammatory cytokines such as TNFα and IL1β cause a release of ET-1 at the BBB level [28-35]. In turn, ET-1 causes the release of other cytokines and chemokines such as MCP-1 [36] thus amplifying the inflammatory signals. ET-1 released in this inflammatory context is known to regulate the P-gp activity. Indeed, long-term exposure of isolated rat brain capillaries to the pro-inflammatory cytokine TNFα caused release of ET-1 and then an increase of P-gp transport activity [31,37]. As inflammation occurs in nearly all CNS disorders [8], it is important to understand how it alters the function of drug efflux transporters since these alterations will affect the efficacy of CNS drugs [37]. However, *in vivo* studies of the impact of ET-1 on the regulation of P-gp and other brain-to-blood ABC transporters such as breast cancer resistance protein (BCRP) in adults and more particularly in children have not been done. From the clinical perspective of developing new drugs with enhanced efficacy in the CNS of children, it is important to understand how the BBB drug efflux transporters are regulated under inflammatory conditions in children’s brains specifically. That is the main target of the present study. We examined and compared regulation of P-gp and BCRP expression and transport activity in young and adult BBB and investigated the molecular mechanisms underlying these processes. To evaluate whether potential developmental differences in neuroinflammatory responses could contribute to the age-specific patterns of BBB efflux transporter expression, rats at postnatal day (P) P21 and P84, corresponding to the juvenile and adult stages of human brain maturation, respectively, were treated with ET-1 by intracerebroventricular (icv) route. Twenty-four hours after ET-1 icv administration, we measured P-gp and BCRP protein expression in isolated brain capillary by immunoblotting, and we assessed their transport activity *in vivo* by measuring the unbound brain/plasma concentration ratios (K_p,uu,brain_) of known efflux transporter substrates [38,39] administered intravenously. The findings are discussed in the context of glial activation leading to differential release of cytokines/chemokines, in particular interleukins-1α, 1-β (IL-1β), -6 (IL-6), -10 (IL-10), monocyte chemoattractant protein (MCP-1/CCL2), fractalkine, and tissue inhibitor of metalloproteinases-1 (TIMP-1). These inflammatory mediators were measured in the brain and serum of rats treated with ET-1 at two stages of brain maturation: P21 (pediatric stage in humans) and P84 (adult stage in humans).

## Methods

### Experimental design

Male Wistar rats at two stages of brain maturation were used in our experiments: Postnatal (P) P21, corresponding to the pediatric stage in human; and P84, corresponding to the adult stage in human. The experimental design was articulated in the following steps: 1) Verification of ventricular stereotaxic coordinates in both juvenile and adult rats; methylene blue was injected intracerebroventricularly in order to visualize the stereotaxic implantation of the injection cannulae and the injection site into the brain lateral ventricles. 2) BBB integrity assessment by the Evan’s Blue test, the baclofen test, and the measure of mRNA relative expression of a tight-junction component, zonula occludens-1 (*ZO-1*); ET-1 was administered intracerebroventricularly. Twenty-two hours later, a solution of 3% Evan’s Blue dissolved in 0.9% saline was administered intravenously (4 ml/kg). Two hours later, brains were isolated and the BBB integrity was assessed by verifying the absence of blue trace and by albumin immunohistochemical (IHC) detection in the cortex and the hippocampus. BBB integrity was further assessed by measuring (using real-time PCR) mRNA relative expression of the *ZO-1* in brain microvessels isolated 24 h after ET-1 treatment. 3) Measure of P-gp and BCRP protein expression in brain microvessels isolated 24 h after ET-1 icv injection. 4) Measure of P-gp and BCRP protein activity *in vivo* 24 h after ET-1 icv injection; Twenty hours after ET-1 icv treatment, P-gp and BCRP substrates were administered intravenously during four hours (steady state (ss) achievement) at a concentration of 1 mg/kg/h. At the end of the infusion period, blood was sampled, brain was isolated and the administered substrates were quantified in the two compartments using mass spectrometry. P-gp and BCRP transport activity was estimated by the ratio between substrates unbound brain concentrations (C) and unbound plasma concentrations. This ratio is described by the unbound partitioning coefficient,

(1) $${\text{K}}_{\text{p},\text{uu},\text{brain}}={{\text{C}}_{\text{ss}}}_{\text{tot},\text{brain}}{\text{x f}}_{\text{u},\text{brain}}/{{\text{C}}_{\text{ss}}}_{\text{tot},\text{plasma}}{\text{x f}}_{\text{uplasma}}\text{.}$$

**Scheme 1:**
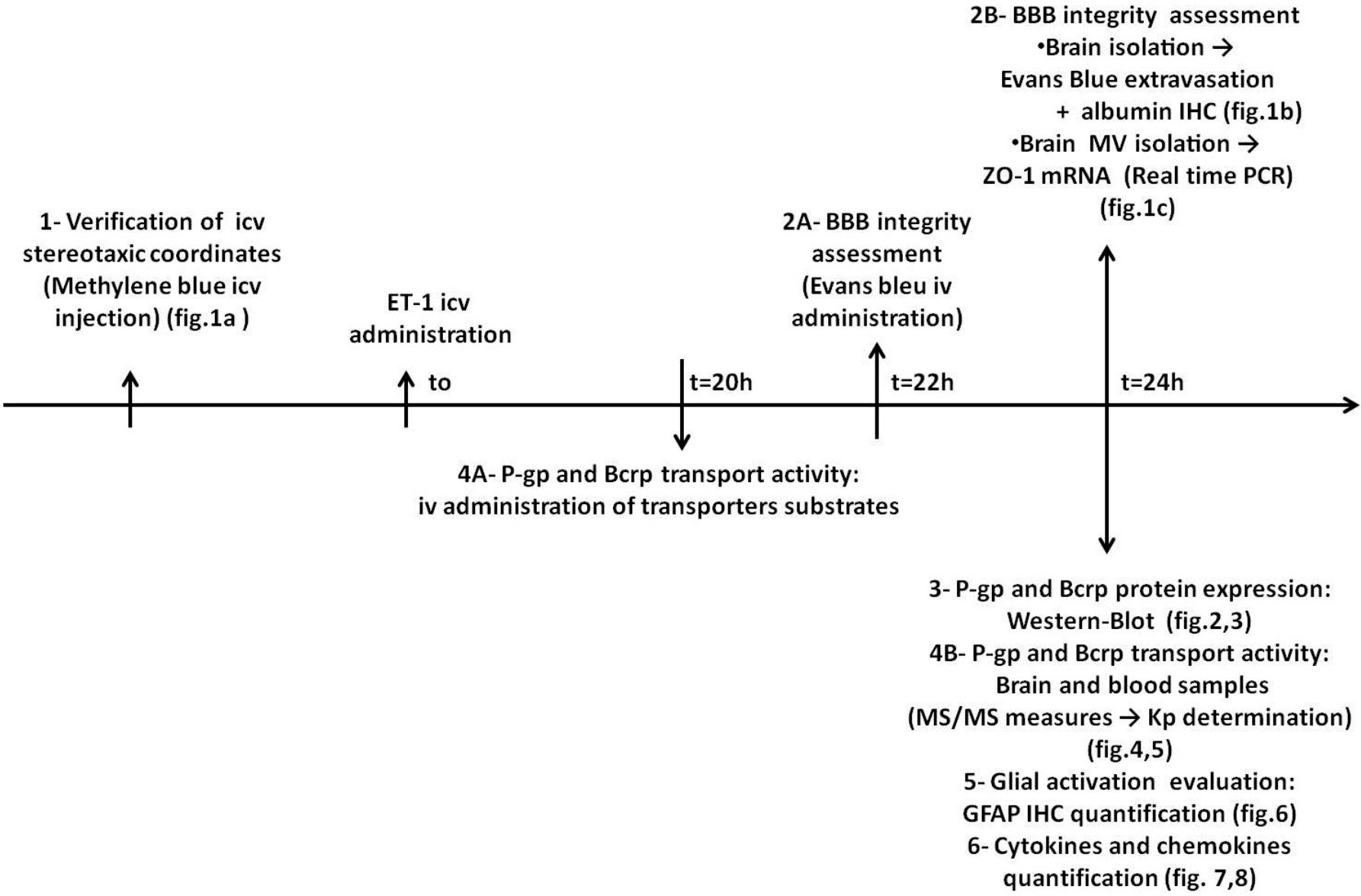
**An illustration of the experimental design. 1**. Verification of intracerebroventricular (icv) stereotaxic coordinates. **2**. Blood–brain barrier (BBB) integrity assessment. **3**. P-glycoprotein (P-gp) and breast cancer resistance protein (BCRP) expression measurement. **4**. P-gp and BCRP transport activity measurement. **5**. Glial activation evaluation by glial fibrillary acid protein (GFAP) immunohistochemistry. **6**. Brain and serum cytokines/chemokines quantification.

5) Immunohistochemistry of glial fibrillary acidic protein (GFAP), a glial activation marker [40], in cortex and hippocampus of rat brains isolated 24 h after ET-1 treatment. 6) Brain and serum cytokines/chemokines quantification 24 h after ET-1 icv injection. The design of our research methodology is illustrated in Scheme 1.

### Reagents

Bovine serum albumin (BSA), N-alpha-tosyl-L-lysinyl-chloromethylketone (TLCK) and (4-(2-hydroxyethyl)-1-piperazineethanesulfonic acid (HEPES) were from Sigma (Saint Louis, MO, USA). Hank's buffered salt solution (HBSS), phosphate buffered saline (PBS) and penicillin-streptomycin-neomycin (PSN) were from Invitrogen (Carlsbad, CA, USA). Collagenase/dispase and DNase I was from Roche (Indianapolis, IN, USA). The NP-40 reagent (IGEPAL; CA-630) was from Sigma-Aldrich (St-Quentin-Fallavier, France). Primary antibodies from Santa Cruz Biotechnologies (Santa Cruz, CA, USA) were: anti-MDR1 (sc-55510), anti-BCRP (sc-130933). Anti-actin (Mab 1501) was from Millipore (Molsheim, France). The secondary antibodies: goat anti-rabbit, goat anti-mouse and mouse anti-goat IgGs conjugated to horseradish peroxidase (HRP) were from Santa Cruz Biotechnologies.

Digoxin (CAS 20830-75-5) and prazosin (CAS 19237-84-4) were from Sigma-Aldrich (St-Quentin-Fallavier, France). Internal standards vinblastine (CAS 143-67-9) and chlorpropamide (CAS 94-20-2) were from Sigma-Aldrich (Saint-Quentin-Fallavier, France). ET-1 was from Bachem (Heidelberg, Germany).

### Animals

Rats were from Janvier Laboratories (Le Genest St Isle, France). Two groups of male Wistar rats [postnatal days 21 and 84] corresponding to the juvenile and adult stages of BBB maturation, respectively were used. Rats were maintained in a temperature and humidity controlled (19 C-23°C) room under a 12:12 h light–dark cycle, and were fed a standard diet (rodent rat chow) *ad libitum* with free access to tap water. Animal use in this research was approved in accordance with the Declaration of Helsinki, the European community’s council directives (86/609/EEC, November 24, 1986) and the French directives concerning the use of laboratory animals.

### Stereotaxic injections

Rat of different ages were weighed using a digital scale. All animals were then anesthetized with ketamine (87 mg/kg)/xylazine (13 mg/kg) and placed in a rodent stereotaxic apparatus. Skin and cranial fascia were resected and the skull exposed. A injection cannulae (from Cortat SA, Courrendlin, Switzerland) (length 25 mm, outer diameter 0.28 mm, internal diameter 0.18 mm) was inserted into the right lateral cerebral ventricle (4 mm ventral to the dura) via a hole that was drilled in the cranium at 1.08 mm posterior and 2 or 1.5 mm lateral to the bregma for P21 or P84 rats, respectively. The tip of a Hamilton syringe needle was briefly lowered to a depth of 3.8 or 4.2 mm for P21 or P84 rats, respectively, and immediately raised by 0.2 mm to create a trough. Each rat, received a double injection of ET-1 at a dose of 25 pmol/kg, or a vehicle control ((saline solution (0.9% NaCl)). The needle was left in for 5 minutes following the end of the injection, after which it was raised slowly, craniotomies were filled with gel-foam, and the scalp was sutured with wound clips. The wound was closed with self-dissolving sutures and swabbed with iodine. For pain control, lidocaine solution was applied. Animals were placed in a heated recovery cage, where they remained until the end of the study. Twenty-four hours after intracerebroventricular (icv) injection, animals were used for the assessment of unbound brain/plasma concentration of efflux drug substrates (see below) and/or euthanized by anesthetic injection and decapitated. Serum and brains were collected and frozen for further investigations.

### Assessment of blood–brain barrier integrity

The integrity of the BBB was assessed by assessing the brain uptake of Evans blue dye and by following the expression of tight junctions on the brain microvessels. For that, ET-1 was administered icv. Twenty-two hours later, a solution of 3% Evans Blue dissolved in 0.9% saline was administered intravenously (iv, 4 ml/kg). Evans blue binds to serum albumin, giving rise to a high molecular complex which remains in intravascular spaces and diffuses to the extravascular space with BBB disruption [41]. Two hours after Evans blue iv administration, brains were isolated and the BBB integrity was assessed by verifying the absence of blue trace and by albumin IHC detection in the cortex and the hippocampus. BBB integrity was further assessed by measuring (using real-time PCR) mRNA relative expression of a tight-junction component, the *ZO-1*, in brain microvessels isolated 24 h after ET-1 treatment.

### Isolation of brain microvessels

Rat brain microvessels were isolated as described previously [42]. The purity of collected brain microvessels was checked after RNA isolation and RT- PCR experiments by measuring the expression of cell-specific marker genes using specific primer for brain endothelial cells (CD31 or PECAM), for astrocytes (glial fibrillary acid protein or GFAP) and for pericytes (α-actin) as previously described [42].

To further evaluate changes in BBB integrity, the mRNA expression profile of the tight junction zonula occludens-1 (*ZO-1*) was assessed as described previously [5,42]. Quantitative expression of tight junction components was determined using 0.4 μM of cDNA for each primer set in the RT^2^ Pathway-Focused Profiler^TM^ Array from SABiosciences (TJ: catalog CAPR09279) (Frederick, Maryland, USA) according to the manufacturer’s recommended protocol. The specific amplification conditions were 2 minutes at 50°C, 10 minutes at 95°C followed by 40 amplification cycles at 95°C for 0.5 minute and 60°C for 1 minute to reinitialize the cycle again. The specificity of each reaction was also assessed by melting curve analysis to ensure the presence of only one product. Relative gene expression values were calculated as 2^-ΔCT^, where ΔCT is the difference between the amplification curve (CT) values for genes of interest and the housekeeping gene (hypoxanthine-guaninephosphoribosyltransferase, *HPRT*; glyceraldehyde phosphate dehydrogenase, *GAPDH*). If the CT was higher than 35, we considered the expression level too low to be applicable.

### Western blot analysis

To analyze expression at the protein level, western blot analysis was performed on rat cerebral microvessels as reported elsewhere [31,37]. Optical density was quantified using VersaDoc analysis software (BioRad Laboratories, Hercules, CA).

### GFAP immunohistochemistry

Rats were anesthetized with isoflurane and subjected to intra-cardiac perfusion with 0.1% phosphate buffered saline (PBS). The brains were quickly removed and fixed in 4% paraformaldehyde (PFA) for 4 days, incubated in 30% sucrose solution, frozen in isopentane at −50°C, and stored at −80°C. Using a cryostat microtome, 40 μm sagittal brain slices were obtained and stored at −20°C in cryoprotectant solution until processing as free-floating sections. Brain slices were incubated in 30% H_2_O_2_ for 20 minutes before addition of normal goat serum blocking solution. Sections were incubated overnight (12 h) at 4°C with the primary antibody anti-GFAP (rabbit antibody, Abcam ab7260, 1/5000) (Paris, France) then for 30 minutes with the secondary biotinylated antibody (anti-rabbit IgG, ABC kit Abcam ab8627, 1/200). Sections were incubated with an avidine biotine solution (ABC kit Abcam ab 8627) for 30 minutes, and staining was revealed after 8-minute incubation with 3,3’-diaminobenzidine tetrahydrochloride (Sigma), 30% H_2_O_2_. Sections were washed in PBS to stop the reaction.

GFAP image analysis was performed using Visilog 6.8 imaging software (Noesis, France), by measuring the percentage of stained surface over a manually defined area (optical density), excluding the interface of adjacent tissues (10× and 40× objectives). GFAP was assessed in the cortex and hippocampus.

### Brain and serum cytokines/chemokines quantification

#### Protein extraction from brain tissue

Brain samples were weighed and homogenized with a Precellys 24 tissue homogenizer (Bertin Technologies, Montigny-le-Bretonneux, France) in 2 ml tubes containing 1.4 mm of ceramic beads (Cat 03961-1-003, Bertin Technologies). Then 500 μL of lysis buffer (20 mM TrisHCl pH 7.4, 0.15 M NaCl, 2 mM EDTA, 1 mM EGTA with protease inhibitor cocktail (Santa Cruz Biotechologies, sc-29131)) was added to each tube. Samples were centrifuged (100 g) for 10 minutes at 4°C, and then the supernatant was removed and centrifuged a second time (20,000 g for 40 minutes at 4°C) to remove any remaining debris. Protein levels for all samples were quantified by the Bradford method and stored at −80°C.

##### *Measurement of cytokines and chemokines in serum and brain supernatants*

All cytokines, IL-1α, IL-1β, IL-6, IL-10, MCP-1/ccl2, fractalkine, and TIMP-1were simultaneously measured in brain and serum samples using the Agilent technology cytokine microarray (Tebu-bio, Le Perray-en-Yvelines, France). Data were calculated by generating a calibration curb obtained using reconstituted cytokine standard in matrix of serum and buffer for brain samples. After incubation of samples with antibody cocktail and specific wash steps (as per manufacturer), 80 μL of Cy3 equivalent dye-conjugated streptavidin was added to each sample before incubation overnight at 4°C. The signals were visualized with a high-resolution microarray scanner (Agilent Technologies, Cat G2505B). Data extraction was done with the microarray analysis software GenePix 6.0. Quantitative data analysis was done with Quantibody® Q-Analyzer software (RayBiotech, Norcross, GA). All brain values were corrected for individual serum measurements as follows and described previously [6].

(2) $$Brain\;cytokine\left(pg/mg\right)=Brain\;cytokine\left(pg/mg\right)-\left[Serum\;cytokine\left(pg/mg\right)*0.02\%\left(correction\;factor\right)\right]$$

### Assessment of unbound brain/plasma concentration ratio (K_p,uu,brain_) of known efflux transporter substrates

A time-course distribution study of drug in plasma and brain of rats treated or not by pharmacological agents was conducted as previously described [42]. Substrates of specific efflux transporters (digoxin (P-gp substrate) and prazosin (BCRP substrate)) were infused via the femoral vein for 4 h, a time sufficient to achieve steady state, at a concentration of 1 mg/kg/h. Six rats per treatment were used. At the end of the infusion period, blood was sampled from the abdominal aorta in Eppendorf tubes (Eppendorf, Le Pecq, France) containing a sodium heparinate evaporated solution at 1000 U/ml, and then centrifuged for 5 minutes at 3000 g at 4°C to collect plasma. After blood collection, brains were collected and weighed. Plasma samples and brains were stored at −20°C, for subsequent bioanalysis by mass spectrometry.

#### Transporter substrate quantification in biological samples

Brains were mixed with ultrapure water (2 ml/g of brain) using an Ultraturrax T65 system (IKA-Werke, Staufen, Germany) Extract suspensions (200 μL) were submitted to protein precipitation with 1 ml of methanol previously spiked with internal standard (1 μg/ml). After evaporation of the methanolic extracts to dryness, the dried extracts were resuspended in 500 μL of 1% NH_4_OH. Plasma (200 μL) was diluted with 200 μL of 1% NH_4_OH. Both brain extracts and diluted plasmas were submitted to solid–liquid extraction (SPE) on Oasis cartridges (reference 186000366 (Waters, Saint Quentin, France) as previously described [36]. The eluates (2 × 250 μL) of 2% formic acid in methanol and 2 × 250 μL of acetonitrile/methanol (1/1, v/v)) were evaporated to dryness, reconstituted in 100 μL of 10 mM ammonium acetate/acetonitrile/formic acid, 95/5/0.1 v/v and 20 μL was injected into the chromatographic system. Chromatography was performed using a Waters Acquity UPLC system on a BEH Shield RP18 column (2.1 mm × 100 mm, 1.7 μm) coupled with a BEH Shield RP18 1.7 μm Van Guard™ Pre-Column (Waters, Saint-Quentin-en-Yvelines, France). Mobile phase solvent A comprised 0.1% formic acid in 10 mM ammonium acetate, and mobile phase solvent B comprised 0.1% formic acid in acetonitrile. The run time was 5 minutes, and analytes were eluted with the following gradient: from 0 to 1 minute 5% solvent B, from 1 to 2.5 minutes the proportion of solvent B increased linearly from 5 to 80%, from 2.5 to 3 minutes a steady state of 80% solvent B was maintained, and from 3.1 to 5.0 minutes there was re-equilibration of initial conditions. The flow rate was set at 0.6 ml/min, the column temperature was maintained at 60°C and the autosampler at 4°C. The detection was performed with triple quadrupole mass spectrometer Quattro Premier XE monitored by MassLynx software, version 4.1 (Waters, Saint-Quentin-en-Yvelines, France) equipped with an electrospray ionization source operating alternatively in positive and negative mode. Tuning parameters were: capillary voltage 3 kV, source temperature 120°C, and desolvation temperature 350°C. The multiple reaction monitoring transitions for analytes were as follows: m/z digoxin 325.2 >779.4 and m/z prazosine 384.1 >247.2.

Analytes were quantified by means of calibration curves using vinblastine or chlorpropamide as the internal standard.

For plasma assay, calibration ranges were from 1.0 to 200 ng/ml for digoxin and from 0.4 to 170 ng/ml for prazosine. For brain extract assay, calibration ranges were from 1.0 to 100 ng/ml for digoxin and from 0.5 to 100 ng/ml for prazosine.

#### Measurement of the unbound partitioning coefficient of the drugs

The ratio between brain concentrations and plasma concentrations to estimate the delivery of drug to the brain was calculated [39,43]. This ratio can be described by the partition coefficient, *K*_p_:

(3) $$K_{p,brain}=C_{ss_{tot,brain}}/C_{ss_{tot,plasma}}$$

where C_SStot_ is the steady-state drug concentration for total (bound and unbound) in brain and plasma.

Taking into account protein binding in plasma and brain tissue components of efflux/influx transporter substrates (digoxin and prazosin) measured using equilibrium dialysis in triplicate for each compound as described elsewhere (Table 1) [39], the unbound partitioning coefficient *K*_p,uu_ which determined the net influx and efflux of drug across the BBB, was calculated as follows:

(4) $$K_{p,uu,brain}=C_{ss_{tot,brain}}xf_{u,brain}/C_{ss_{tot,plasma}}xf_{\mathrm{upplasma}}$$

where *f*_u,plasma_ and *f*_u,brain_ is the fraction of unbound drug (Table 1) in plasma and brain tissue, respectively.

**Table 1:** **Digoxin and prazosin unbound protein fraction in plasma (f**_**u,plasma**_**) and brain tissue (f**_**u,brain**_**)**

**Compounds**	**f**_**u,plasma**_	**f**_**u,brain**_	**f**_**u,brain**_**/f**_**u,plasma**_**ratio**
Prazosin	0.204 ± 0.001	0.071 ± 0.004	0.349
Digoxin	0.379 ± 0.001	0.218 ± 0.027	0.576

### Statistical analysis

Statistical analysis was performed using the Prism 3.0 program (GraphPad Software, Inc, San Diego, CA) and the R statistical software (R Development Core Team, 2009). Comparisons between groups were performed using one-way analysis of variance (ANOVA) with the Student-Bonferroni-post test and the unpaired Student’s *t* test. Data are presented as mean ± S.E.M. Statistical significance was set at *P* <0.05.

## Results

### Blood–brain barrier integrity assessment after intracerebroventricular injection of endothelin-1 in rats

**Figure 1:**
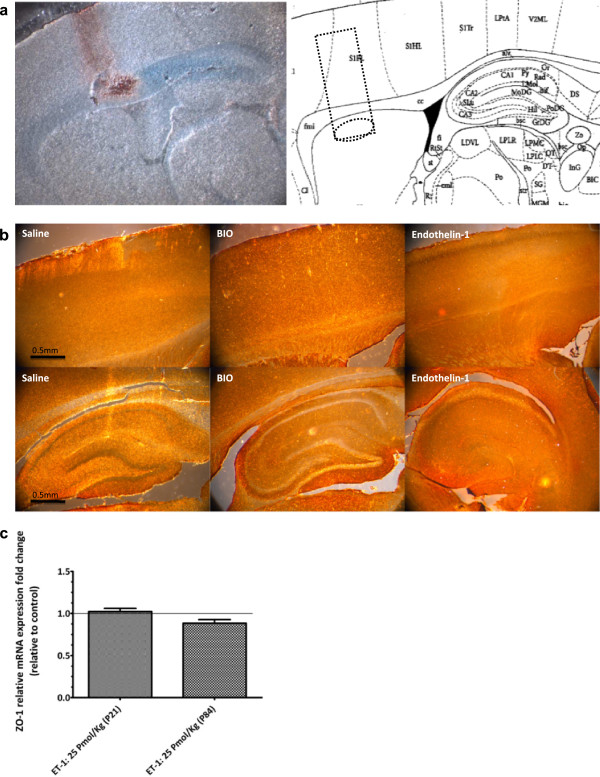
**Representative immunohistochemical staining of albumin in the adult and juvenile brains (scale bars, 5 μm).A**) Stereotaxic implantation of the cannulae was checked using intracerebroventricular (ICV) methylene blue-injected rats which permitted visualization of the stereotaxic implantation of the injection cannulae and the injection site into the brain lateral ventricles. Cannula implantation reached the lateral ventricle as shown by the blue color and cannula scare (blood trace) (as referred to in Paxinos and Watson’s stereotaxic atlas). Moreover, methylene blue diffused to the hippocampus. **B**) Blood–brain barrier (BBB) permeability after endothelin-1 (ET-1) injection was assessed using albumin immunohistochemical (IHC) in IV Evans blue injected rats. ET-1 was administered intracerebroventricularly. Twenty-two hours later a solution of 3% Evans Blue dissolved in 0.9% saline was administered intravenously (4 ml/kg). No albumin labeling or blue trace was detected in the cortex or in the hippocampus of adult or juvenile rat brains, suggesting a lack of BBB breakdown after icv injection of ET-1. **C**) BBB permeability after ET-1 injection was also assessed by measuring (using real-time PCR) mRNA relative expression of a tight-junction component, the *ZO-1* gene, in brain microvessels isolated 24 h after ET-1 treatment. Results represent mean ± S.E.M from three batches of brain endothelial microvessels. Statistical comparisons: *P <0.05, **P <0.01 and ***P <0.0001.

An intact BBB simplifies the assessment of BBB-efflux transporter activity. We used Evans blue to determine whether ET-1 in our experimental conditions altered BBB integrity in juvenile or adult rats. Results depicted in Figure 1 demonstrate that in control animals as well as in ET-1-treated animals no albumin labeling or blue trace was detected in the cortex or in the hippocampus. These results showed no change in BBB permeability. Moreover, there was also no change in the quantitative real-time PCR analysis of *ZO-1* mRNA from freshly isolated rat brain microvessels after ET-1 treatment. Together, these results suggest that ET-1 under the conditions of these experiments does not change BBB permeability.

### Comparison of P-glycoprotein and breast cancer resistance protein expression in juvenile and adult brain microvessels during brain inflammation mediated by endothelin-1

**Figure 2:**
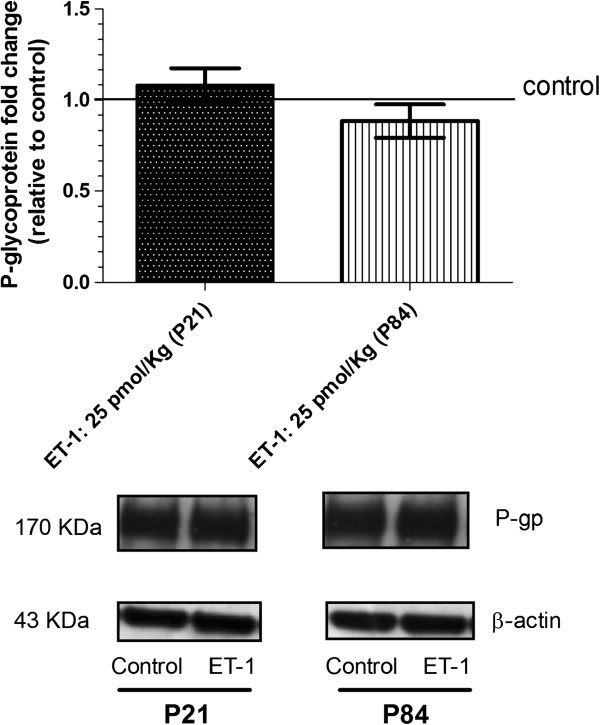
**Expression of P-glycoprotein in freshly isolated rat brain microvessels after endothelin-1 treatment of juvenile and adult rats.** Rats equivalent to pediatric (P21) and adult (P84) human brain were used. Twenty-four hours after intracerebroventricular (icv) injection of endothelin-1 (ET-1) was used to trigger inflammatory reactions, brain microvessels were isolated, and P-glycoprotein expression was assessed by western blot as reported in Methods. Results show no difference in P-glycoprotein expression after ET-1 treatment. Immunoblot represents mean ± S.E.M from three batches of brain endothelial microvessels. Statistical comparisons: *P <0.05, **P <0.01 and ***P <0.0001.

**Figure 3:**
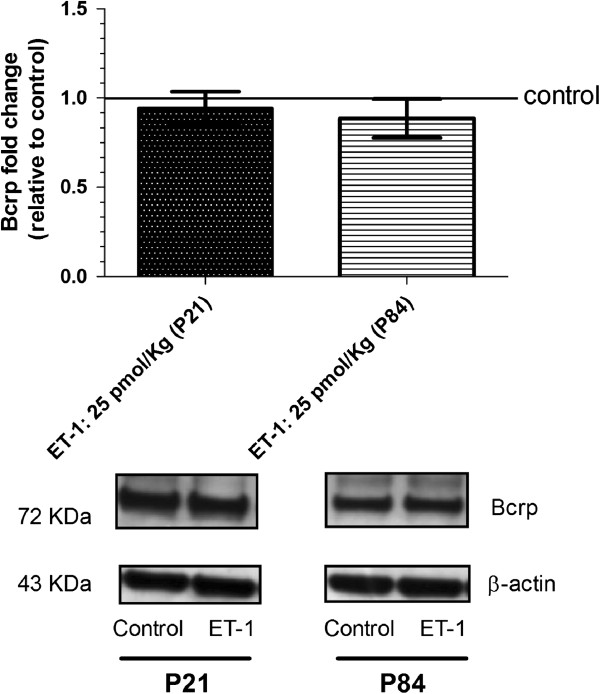
**Expression of breast cancer resistance protein (BCRP) in freshly isolated rat brain microvessels after endothelin-1 treatment of juvenile and adult rats.** Rats equivalent to pediatric (P21) and adult (P84) human brain were used. Twenty-four hours after intracerebroventricular (icv) injection of endothelin-1 (ET-1) was used to trigger inflammatory reactions, brain microvessels were isolated, and BCRP expression was assessed by western blot as reported in Methods. Results show no difference in BCRP expression after ET-1 treatment. Immunoblot represents mean ± S.E.M from three batches of brain endothelial microvessels. Statistical comparisons: *P <0.05, **P <0.01 and ***P <0.0001.

Previous *in vitro* results suggest that short-term exposure of adult rat brain endothelial cells to ET-1 decreases P-glycoprotein [31], whereas longer-term exposure (24 h) of adult human brain endothelial cells causes no changes in P-glycoprotein expression [7]. We found here that ET-1 administration into brain did not alter P-gp (Figure 2) or BCRP (Figure 3) expression in adult or in juvenile brain microvessels isolated twenty-four hours after ET-1 administration, as revealed by western blot.

### Comparison of transporter activity in juvenile and adult blood–brain barrier during brain inflammation mediated by endothelin-1

**Figure 4:**
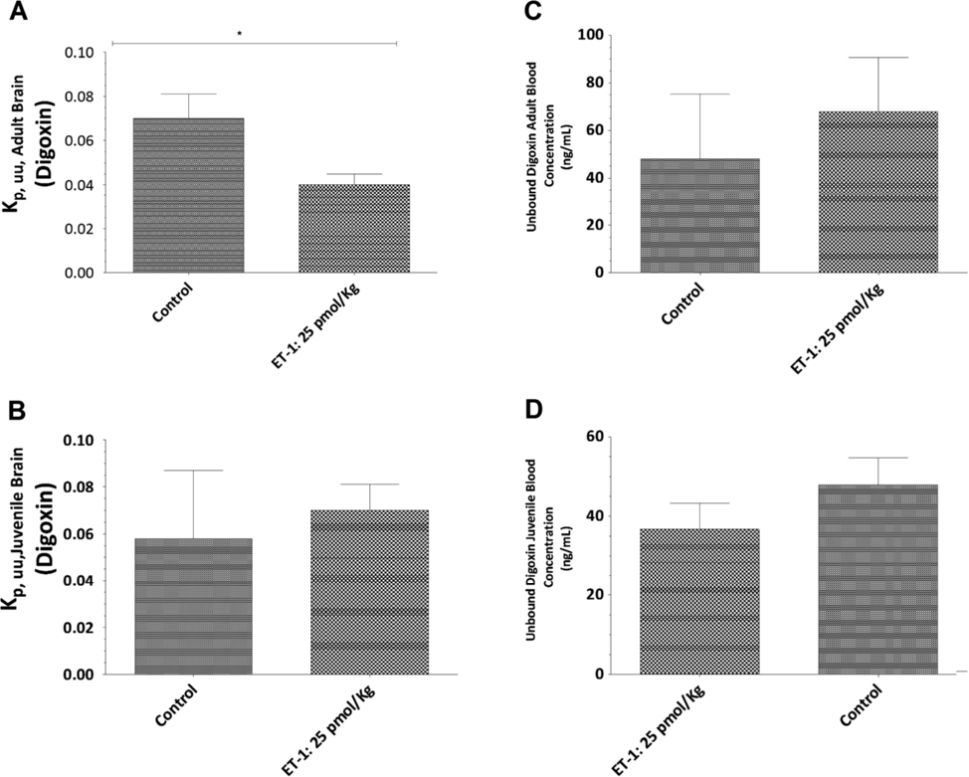
**P-glycoprotein activity regulation at the blood–brain barrier through activation of endothelin-1 signaling.** Twenty-hours after endothelin-1 (ET-1) intracerebroventricular (icv) treatment, digoxin, a P-glycoprotein (P-gp) substrate, was administered intravenously in adult brain (**A**) and juvenile brain (**B**) during four hours (steady state (ss) achievement) at a concentration of 1 mg/kg/h. At the end of the infusion period, blood was sampled, brain was isolated and the administered substrate was quantified using mass spectrometry. P-gp transport activity was estimated by the ratio between the substrates’ unbound brain concentrations and unbound plasma concentrations_._ Unbound plasma digoxin concentration in adult (**C**) and juvenile rat (**D**) was also calculated. ET-1 induces an enhanced transporter activity of P-gp at the adult blood–brain barrier (BBB), whereas no significant change occurred in P-gp activity at the juvenile BBB. Data represent the mean concentration for five rat brains/plasma. Variability is given by bars ± S.E.M. Data were analyzed using Student’s *t*-test. Statistical comparisons: *P <0.05.

**Figure 5:**
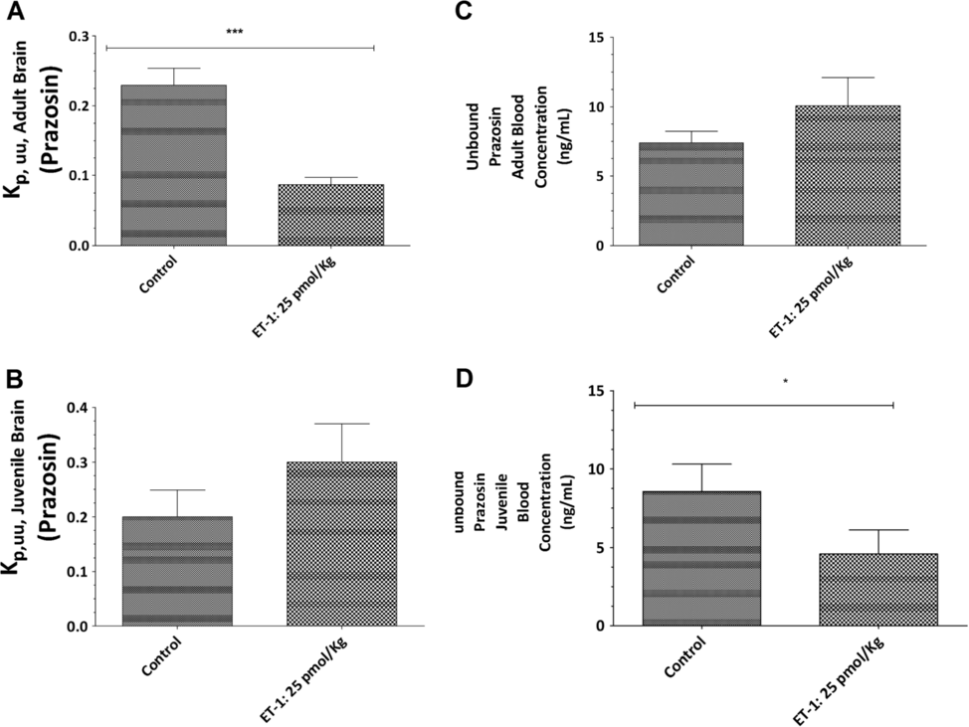
**BCRP activity regulation at the blood–brain barrier through activation of endothelin-1 signaling.** Twenty-hours after endothelin-1 (ET-1) intracerebroventricular (icv) treatment, prazosin, a BCRP substrate, was administered intravenously in adult brain (**A**) and juvenile brain (**B**) during four hours (steady state (ss) achievement) at a concentration of 1 mg/kg/h. At the end of the infusion period, blood was sampled, brain was isolated and the administered substrate was quantified using mass spectrometry. BCRP transport activity was estimated by the ratio between unbound prazosin brain concentrations and unbound plasma prazosin concentrations. Unbound plasma prazosin concentration in adult (**C**) and juvenile rat (**D**). Data represent the mean concentration for three or five rat brains/plasma. Variability is given by bars ± S.E.M. Data were analyzed using Student’s *t*-test. Statistical comparisons: *P <0.05.

To determine whether there were differential responses to ET-1 in transporter activity of P-gp and BCRP in the juvenile and adult BBBs, the unbound brain and unbound plasma concentration ratios (K_p,uu,brain)_ for known substrates of P-gp (digoxin) and BCRP (prazosin) were determined 24 h after ET-1 icv injection. In the untreated state, there were no differences between juveniles and adults for P-gp (Figure 4) or BCRP (Figure 5) activity. However, ET-1 used to trigger neuroinflammatory reactions characterized by cytokines/chemokines secretion, caused different regulation of P-gp and BCRP transport activity (Figures 4 and 5) between juvenile and adult rats. Notably, ET-1 induced an enhanced transporter activity of P-gp as assessed by digoxin efflux from brain [K_p,uu,brain_ = 0.04 ± 0.006, for treated adult rat *versus* K_p,uu,brain_ 0.07 ± 0.01, *P* <0 .05), whereas no significant change occurred in P-gp activity at the juvenile BBB (Figure 4). The decrease of brain uptake in the ET-1-treated adult animals strikingly coincided with the increases in the unbound plasma concentration of digoxin compared with the control (67.84 ± 22.74 ng/ml *versus* 47.75 ± 10.30 ng/ml), whereas no significant change in the plasma concentration of digoxin compared with the control was observed in juvenile rats (36.76 ± 6.50 ng/ml *versus* 47.75 ± 6.79 ng/ml). ET-1 also enhanced efflux of prazosin, the substrate for BCRP, at the adult BBB [K_p,uu,brain_ = 0,09 ± 0.01 , for treated adult rat *versus* K_p,uu,brain_ 0.23 ± 0.02, *P* <0.0001)], with a corresponding arithmetic increase in unbound plasma concentration (10.06 ± 2.03 *versus* 7.40 ± 0.90 ng/ml) (Figure 5). Conversely, at the juvenile BBB, 24 h after ET-1 treatment, efflux of prazosin tend to decrease [K_p,uu,brain_ = 0.30 ± 0.07, for treated adult rat *versus* K_p,uu, brain_ 0.19 ± 0.05, for animal controls (*P* = 0.11)]. This tendency to decrease in BCRP activity at the juvenile BBB was strikingly associated with decreased unbound plasma prazosin concentration (4.60 ± 1.52 ng/ml *versus* 8.56 ± 1.75 ng/ml, *P* <0 .05) (Figure 5).

### Comparative glial activation between juvenile and adult rat brain cortex and hippocampus under inflammation mediated by endothelin-1

**Figure 6:**
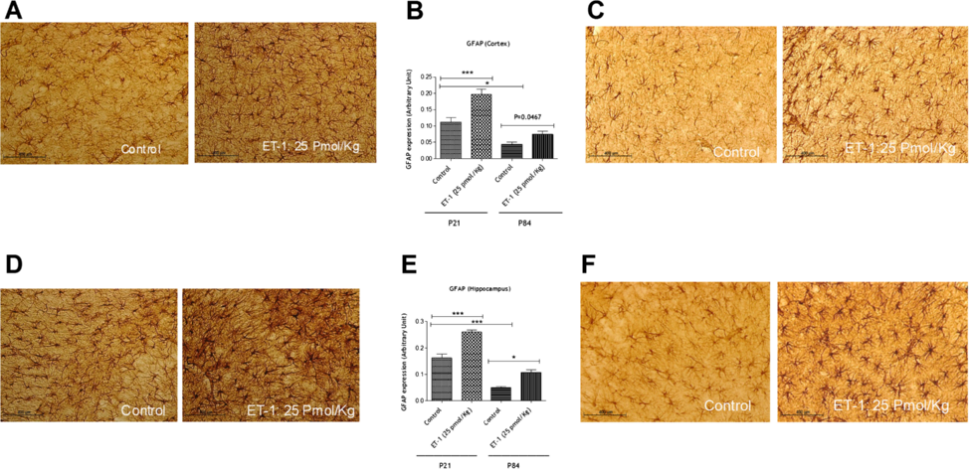
**Effect of endothelin-1 on glial activation in adult and juvenile brains.** Representative immunohistochemical staining of glial fibrillary acidic protein (GFAP) in the juvenile (**A**) and adult (**C**) brains cortex and quantitative analysis of GFAP expression (**E**). Comparisons between groups were performed using one-way analysis of variance (ANOVA) with Bonferroni post test. Data are presented as mean ± S.E.M. Statistical significance was set at *P* <0.05. Each bar is expressed as the mean ± S.E.M; ***P <0 .0001 compared with control group. (**D**) Representative immunohistochemical staining of GFAP in the juvenile brain and adult (F) hippocampus and quantitative analysis of GFAP expression (**E**). Each bar is expressed as the mean ± S.E.M; ***P <0.0001 compared with control group.

We determined whether the activation of glial cells by ET-1 in juvenile and adult rat brains could be responsible for the differential effects of ET-1 on efflux transporter activity. Immunohistochemistry depicted in Figure 6 shows that ET-1 induced activation of glial cells as measured by GFAP. In cortex, ET-1 increased GFAP in glial cells in adults (1.94-fold,) as well as in juvenile brain (1.78-fold, *P* <0.0001). However, variance analysis (one-way ANOVA) with Bonferroni’s multiple comparison post test showed difference in the basal glial activation between juvenile and adult brain cortex (*P* = 0.0467). In the hippocampus, the basal glial activation is statistically different between juvenile and adult (*P* = 0.0013), but ET-1 enhanced GFAP expression in glial cells of adult brain 2-fold and in juvenile brain 1.62-fold (Figure 6).

### Cytokine and chemokine responses mediated by endothelin-1 in juvenile and adult brains

**Figure 7:**
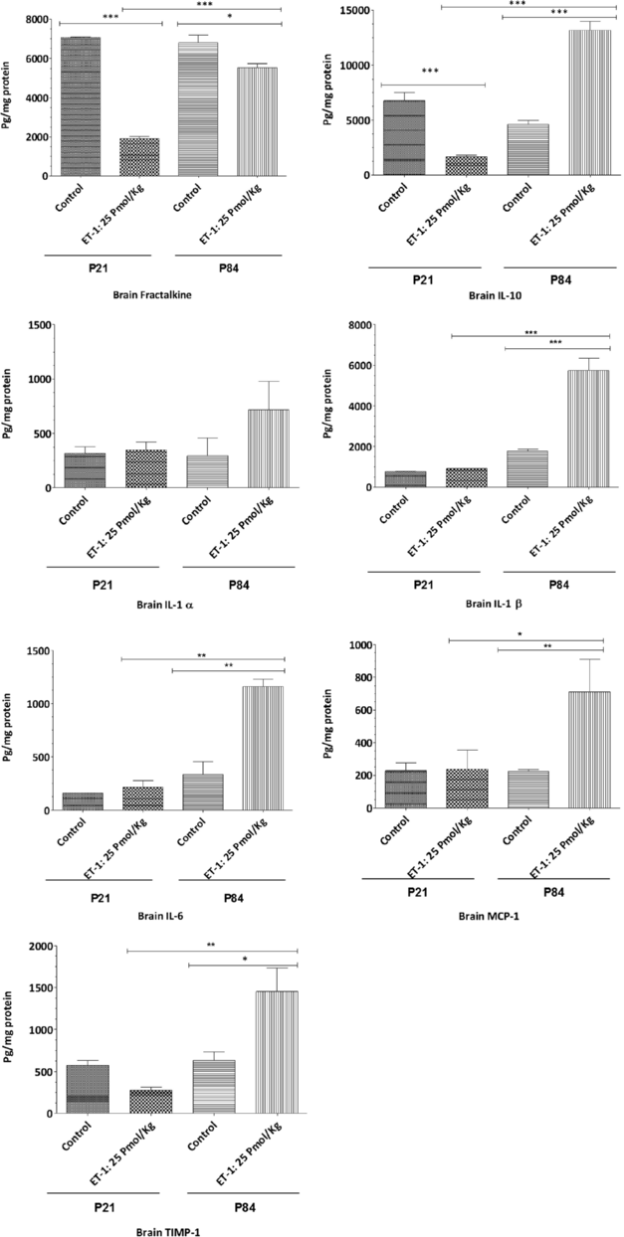
**Cytokines and chemokines levels for 24 hours in juveniles (P21) and adult (P84) rat brains after a single intracerebral injection of endothelin-1.** All cytokines, IL-1α, IL-1β, IL-6, IL-10, MCP-1/ccl2, fractalkine and TIMP-1 were simultaneously measured in brain and serum samples using the Agilent technology cytokine microarray (Tebu-bio). All brain values were corrected for individual serum measurements as described previously [6]. Comparisons between groups were performed using one-way analysis of variance (ANOVA) with the Student-Bonferroni post test. Data are presented as mean ± S.E.M. Statistical significance was set at *P* <0.05. Graph plotted as mean ± S.E.M. *P <0.05, **P <0.01, ***P < 0.0001 compared with animal controls. The number of animal tested: n = 3 or 5 rats for each group.

Cytokines and chemokines were measured in brain and serum 24 h after ET-1 injection (25 pmol/kg). Protein expression of cytokines and chemokines in the brain was calculated taking into account that 0.02% of the analyte serum value for serum is predicted to contribute to the value for the brain [6]. Expression patterns of all cytokines and chemokines are depicted in Figure 7 for juvenile and adult rat brain, respectively. Among the panel of cytokines and chemokines tested, only IL-10, IL-6, CCL2/MCP-1, IL-1α, IL-1β, fractalkine, and TIMP-1 were detected. Cytokines that remained significantly elevated in adult brain were IL-10, Il-1β, IL6, Ccl2/MCP1 and TIMP-1. Adult and juvenile rat brains showed different patterns for fractalkine, TIMP-1, IL-10, Il-1β, and Ccl2/MCP-1. The expression of fractalkine, IL-10 and TIMP-1 was significantly decreased in juvenile brain, but increased in adult brain. In addition, we noted a striking difference in the magnitude of Il-1β, at basal level and after ET-1 administration [3.23-fold increase (*P* <0.001) *versus* 1.22 (*P* <0.01) fold increase), and of IL-6 [3.46-fold increase (*P* <0.05) *versus* 1.34-fold increase] secretion between adult and juvenile brain. IL-1α showed the same pattern in juvenile and adult brain.

## Discussion

CNS pharmacotherapy is impeded by the existence ‘CNS barriers’ at the interface between blood and neural tissue. As a result, many drugs fail as therapeutic agents for the CNS because they are pumped out of the brain. To overcome these drug efflux transporters, recent research in the field aims at identifying the factors and the intracellular signaling mechanisms implicated in their regulation in order to modulate their activity and improve pharmacotherapy of brain diseases. For years, BBB efflux transporters have been studied in the adult organism. But, there is a wide-spread belief among pediatricians, neurologists, and neuroscientists that the BBB in the embryo, fetus, newborn, and infant is ‘immature’, implying caution in giving drugs to infants [44]. Moreover, intricate developmental processes are taking place during the prenatal and postnatal periods, which might mean that BBB efflux transporters could also undergo important changes during brain maturation, and might possibly have age-related differences in the inflammatory response. There is growing evidence suggesting that the immune system, through systemic or cerebral inflammation, disturbs the BBB efflux transporters [45], and these alterations can affect the efficacy of CNS-acting drugs.

However, current knowledge on the functional status of the BBB in immature organism remains very limited. Thus, from the clinical perspective of developing new drugs with enhanced efficacy in both the adult and children CNS, it is important to understand 1) the role of the BBB drug efflux transporters in the CNS at the different stages of brain maturation and 2) the mechanisms that regulate their functional activity, under both normal and inflammatory conditions.

In this study, we evaluated the impact of acute cerebral inflammation mediated by ET-1 on BBB efflux transporters with a comparison between juvenile and adult rats. Specifically, we evaluated, *ex vivo* and *in vivo*, the impact of intracerebroventricular (icv) injection of ET-1, on the expression and activity of two of the most clinically relevant BBB drug efflux transporters, the P-gp and the BCRP transporters.

Our results showed that, under inflammatory conditions, BBB drug efflux transporters are regulated differently in juvenile rats when compared to adult rats, and that this differential regulation may be due, in part, to a maturational difference in glial basal levels, and neuro-inflammatory response triggered by ET-1.

Since inflammation has been reported to influence BBB integrity [27,28], we first investigated in our experimental conditions whether icv injection of ET-1 changed BBB permeability. BBB permeability was evaluated by assessing the brain uptake of Evans blue dye to ensure the functionality of the BBB to macromolecule entry after ET-1 treatment, and by following the expression of tight junctions on the brain microvessels to evaluate the integrity of tight junctions which restrict paracellular movement to small molecules across the BBB. Results depicted in Figure 1 demonstrate a lack of BBB permeability changes with the use of Evan blue as well as the use of baclofen, as small molecule which does not cross the BBB (data not shown). In addition, we found no modification in the *ZO-1* gene expression during the cerebral inflammation triggered by ET-1 treatment, and this in both juvenile and adult brains. These findings allowed us to determine the impact of neuroinflammation on the expression and transport activity of BCRP and P-gp in adult and juvenile brains at the BBB. We first compared P-gp and BCRP transport activity between juvenile and adult BBB, and found that juvenile and adult BBB have the same P-gp and BCRP transport activity (Additional file 1). We also found evidence that the transport activities of P-gp and BCRP are enhanced by icv ET-1 at the adult BBB, whereas no significant modulation of P-gp transport activity and a tendency to decrease in BCRP transport activity were seen at the juvenile BBB (Figures 4 and 5). Thus, these results gave evidence that P-gp and BCRP transporters at the BBB level are regulated differently under pathological conditions in juvenile brain when compared to adult. These findings emphasize the importance of considering differential P-gp and BCRP transporter regulation mechanisms between juvenile and adult BBB in the context of pathological conditions.

Second, after assessing BBB transport activity in adult and juvenile brains in the context of ET-1 treatment, we aimed at understanding the underlying mechanisms behind this differential regulation. We suspected a role of neuroinflammatory response triggered by ET-1 because it is likely that cytokine secretion in adult and juvenile brains controls the regulation of BBB transporters [9]. In the brain, the inflammatory response begins with recruitment of the innate immune system. Rapidly, in response to infection or injury, microglia, major inflammatory cells of the monocyte/microphage lineage that reside in the brain, are activated [46]. Microglia are important phagocytic cells, and once activated they release numerous inflammatory molecules, particularly pro and anti-inflammatory cytokines and chemokines [47] (Proinflammatory molecules such as tumor necrosis factor-α (TNF-α), interleukin-1β (IL-1β), interleukin-6 (IL-6) [48-50], chemokines (IL-8, MIP-1α, MIP-1β, MCP-1) [51,52], proteases [53], and anti-inflammatory molecules such as TGF-β and IL-10 [54]). Later, astrocytes are activated [55]. Apart from being involved in a variety of physiologic processes, astrocytes rapidly react to different neurological insults. Upon activation, a series of changes occur in astrocytes, leading to the acquisition of macrophage differentiation markers and effector properties. One main feature of these changes is the increase in the number and size of glial fibrillary acidic protein (GFAP) expressing cells. GFAP is an intermediate filament cytoskeletal protein expressed primarily by astrocytes and it is considered as the marker of astrocytes [40]. Concomitant with GFAP overexpression, astrocytes release many proinflammatory mediators and upregulate the expression of several inflammatory molecules, contributing to the amplification of inflammation [34]. These facts allow us in this study, to test whether the inflammatory response triggered by ET-1 can be responsible for the differential regulation of P-gp and BCRP transporters between juveniles and adults. For that, we quantified 1) the glial activation marker, the GFAP in hippocampus and cortex of rat brains treated with ET-1, and 2) a panel of cytokines and chemokines that could be induced by ET-1 in both juvenile and adult rat brains. We found a difference in the GFAP basal levels between the two populations either in hypocampus or in the brain cortex, but our results showed a pronounced glial activation in adult and juvenile cortex and hippocampus coincided with cytokine/chemokine levels (Figures 6 and 7). Our findings were that IL6, IL-1β, CCl2/MCP-1, TIMP-1 and IL-10 increased significantly in adult brain compared with juvenile brain. These results emphasize the fact that brain development such as glial maturation is likely of paramount importance in the synthesis of specific cytokines such as IL6 for example. Indeed, IL6 is a well-known marker of glial activation [56] and the autocrine action of this interleukin on glial cells might account for an increase of ABC transporters at the cell surface. This increase is important in the secretion of ccl2/MCP-1 by astrocytes upon toll-like receptor 3 activation as reported recently [57]. Thus, a differential GFAP basal level and a differential secretion of cytokines might lead to a differential regulation of ABC transporters at the BBB level. Moreover, taking into account the differential effect of ET-1 on cytokine levels in juvenile and adult brains, we suggest that the increased levels of cytokines in the adult brain and more particularly IL-6 and IL-1β may cause functional but not transcriptional regulation of P-gp and BCRP in adult BBB. Indeed, there was no modulation of protein synthesis in BCRP and P-gp in adult rat brain microvessels (Figure 5). Modulation of transport activity for P-gp in response to peripheral pain inflammation with no increase in its protein expression has been previously reported [58]. We found no published results addressing whether cerebral inflammation as mediated by ET-1 regulates either P-gp or BCRP activity at the adult or juvenile BBB. Based on our results and the literature, we suggest that cytokine synthesis in the adult brain modulates BCRP and P-gp activity at the BBB by post-translational mechanisms such as phosphorylation and cellular localization of transporters. Inflammation regulates a number of intracellular signal transduction pathways [59-61] involved in the regulation of transporter activity. At the BBB, P-gp has been localized to plasma membrane surfaces as well as several subcellular sites, and there is overwhelming evidence suggesting that the localization of P-gp and its trafficking within brain endothelial cells contributes to its function [62,63]. To formally demonstrate that cytokine synthesis is a cause of the differential regulation of transporter activity between juvenile and adult BBB, we increased the amount of ET-1 administered after determining the lack of BBB breakdown. At the dose of 125 pmol/kg of ET-1, the juvenile brain exhibited increases in IL1β, MCP-1, and IL6 ( Additional file 2). Despite the level of those cytokines in the brain, the juvenile BBB showed a decrease in BCRP activity which strikingly coincided with the decrease of unbound plasma prazosin concentration (3.77 ± 1.58 ng/ml *versus* 8.56 ± 3.77 ng/ml, *P* <0.05). In addition, the increase in the administered dose of ET-1 did not change the activity of P-gp in the juvenile BBB. We observed no significant decrease of digoxin concentration in unbound plasma of juvenile animals (23.50 ± 7.58 treated animals *versus* 47.88 ± 6.80 for control animals, *P* <0.05). In adult brain, BBB at the dose of 125 pmol/kg of ET-1 (data not shown) showed the same profile regarding the increase of P-gp and BCRP activity compared with the dose of 25 pmol/kg. These findings suggest the involvement of other parameters in the differential regulation of P-gp and BCRP at the BBB, particularly in juvenile brain. Thus, further investigation is warranted to define more precisely the underlying mechanisms. It has been reported that the transcriptional activity of ABC transporters is under the control of orphan nuclear receptors such as steroid and xenobiotic receptors, and that their expression and function are regulated by environmental stimuli that induce stress. Recent studies show that increased transporter expression occurs in response to signals that activate specific transcription factors including, PXR, CAR, NF-κB and AP-1, and reduced transporter activity occurs rapidly and reversibly in response to signaling through Src kinase, protein kinase C and estrogen receptors [8]. Moreover, Bauer and colleagues have shown in rat brain capillaries that tumor necrosis factor alpha (TNF-α) binding to its receptor TNFR1 leads to the release of ET-1 which in turn acts through its receptor ETB to continue the signaling cascade via nitric oxide synthase (NOS) and protein kinase C (PKC) [31]. This long-term exposure leads to an increase of P-gp activity [31] which is in agreement with our results regarding the adult brain but not the juvenile brain even at low dose of ET-1 (25 pmol/kg) or at the dose of 125 pmol/kg. Thus, to determine the underlying mechanisms behind the differential regulation of the BBB P-gp and BCRP transporter further studies are required and these must be primarily focused on the role of these multiple signaling pathways modulating the expression and activity of ABC transporters at the BBB level in children compared to adults. In addition, difference in the ontogenesis of ET-1 receptor (ETA) evidenced (data not shown) in our laboratory at the level of BBB in juvenile and adult brain might be also taken into account to explain this differential functional regulation of P-gp and BCRP transporter.

## Conclusions

In the present study, we found that BBB transporter activity during neuroinflammation is not affected in the same manner in juvenile brain as it is in the adult brain. This is the first report that illustrates the differential regulation of BBB transporter activity in pediatric and adult brains. In the pediatric brain long-term exposure to ET-1 leads to no modulation of BBB Pgp transport activity and BCRP transport activity tends to decrease while a significant increase of both transporters activity was evidenced in the adult brain. Further studies such as microglial activation, ontogenesis of endothelin receptors (ETB, ETA), NFkβΙ, ΙΙ, PKC isoforms, NOS, sphingolipid signaling pathways in the context of brain inflammation are warranted to understand accurately and precisely the differential regulation of BBB-ABC transporters in pediatric and adult brains. In this context, we suggest that potential selective reduction of transcriptional factors such as PKC and NOS isoform in the brain cortex could decrease BBB BCRP transport activity and lead to enhancement of CNS drug pharmacotherapy in pediatric brain. Our study gives the evidence of age-related differences in the regulation of drug efflux transporters under inflammatory conditions, and emphasizes the importance of taking into account the specific properties of the juvenile BBB and distinguishing it from the adult one in the clinical perspective of developing new drugs with enhanced efficacy in children’s CNS. Subsequently, it is highly important to determine the pathways modulating the activity of drug efflux transporters under pathological conditions, both in children and adult brains, because targeting these pathways may open new therapeutic avenues to improve drug delivery into the brain.

## Abbreviations

ABC: Adenosine triphosphate-binding cassette; BBB: Blood–brain barrier; BCRP: Breast cancer resistance protein; BSA: Bovine serum albumin; CNS: Central nervous system; CT: Amplification curve; ET-1: Endothelin-1; GAPDH: Glyceraldehyde phosphate dehydrogenase; GFAP: Glial fibrillary acidic protein; HBSS: Hank's buffered salt solution; HEPES: 4-(2-hydroxyethyl)-1-piperazineethanesulfonic acid; HPRT: Hypoxanthine-guaninephosphoribosyltransferase; HRP: Horseradish peroxidase; IHC: Immunohistochemical; MCP: Monocyte chemoattractant protein; icv: Intracerebroventricular; iv: Intravenously; MDR: Multidrug resistance; P: Postnatal day; PBS: Phosphate buffered saline; PFA: Paraformaldehyde; P-gp: P-glycoprotein; PSN: Penicillin-streptomycin-neomycin; SS: Steady state; TIMP-1: Tissue inhibitor of metalloproteinases-1; TLCK: N-alpha-tosyl-L-lysinyl-chloromethylketone; ZO-1: Zonula occludens-1.

## Competing interests

The authors declare that they have no competing interests.

## Authors’ contributions

RH carried out all experiments, stereotaxic injections, data acquisition, analysis, interpretation and participated in the manuscript’s drafting. ASV was involved in analysis and data interpretation. WB and AM have been involved in the conception, rational, drafting manuscript critically and have given final approval of the version published. All authors have read and approved the final manuscript.

## Supplementary Material

Additional file 1 **Age-related changes in P-gp, and bcrp function at the BBB.** Rats at pediatric (P21) and adult (P84) stages of braindevelopment were used. The in vivo plasma and brain exposure of tested efflux transporter substrates [digoxin (P-gp substrate): 0.5 mg/kg h,, and prasozin (bcrp substrate): 0.25 mg/kg h] was assessed using unanesthetized rats catheterized in the femoral vein. Drug infusion was performed for a period of 4 h (steady state), and then the unbound partitioning coefficient brain/plasma concentration ratio (Kpuu, brain) for each tested efflux transporter substrate was determined as described in the Experimental Section. Data represent the mean concentration for 3 or 5 rat brains and 3 or 5 plasmas; variability is given by bars ± SEM. Statistical comparisons: *P < 0.05, **P < 0.01, and ***P < 0.0001. Click here for file

Additional file 2 **Cytokines and chemokines levels in juvenile brain after a single intracerebral injection of endothelin-1 for 24 h at the dose of 125 pmol/kg.** All cytokines, IL-1 β, IL-6, IL-10, MCP-1/ccl2, fractalkine, TIMP-1, IL-13, cinculin (CINC-1) were simultaneously measured in brain and serum samples using the Agilent technology cytokine microarray (Tebu bio). All brain values were corrected for individual serum measurements as follows and described previously [6]: Graph plotted as mean ± S.E.M. *P <.05, **P <0.01, ***P <0.001 compared with animal controls with n = 3 or 5 rats for each group. Click here for file
