# Supplementary figures and images for: Susceptibility of juvenile and adult blood–brain barrier to endothelin-1: regulation of P-glycoprotein and breast cancer resistance protein expression and transport activity
Source: J Neuroinflammation. 2012 Dec 19;9:273. doi: 10.1186/1742-2094-9-273 (PMC3547749; doi:10.1186/1742-2094-9-273)

**
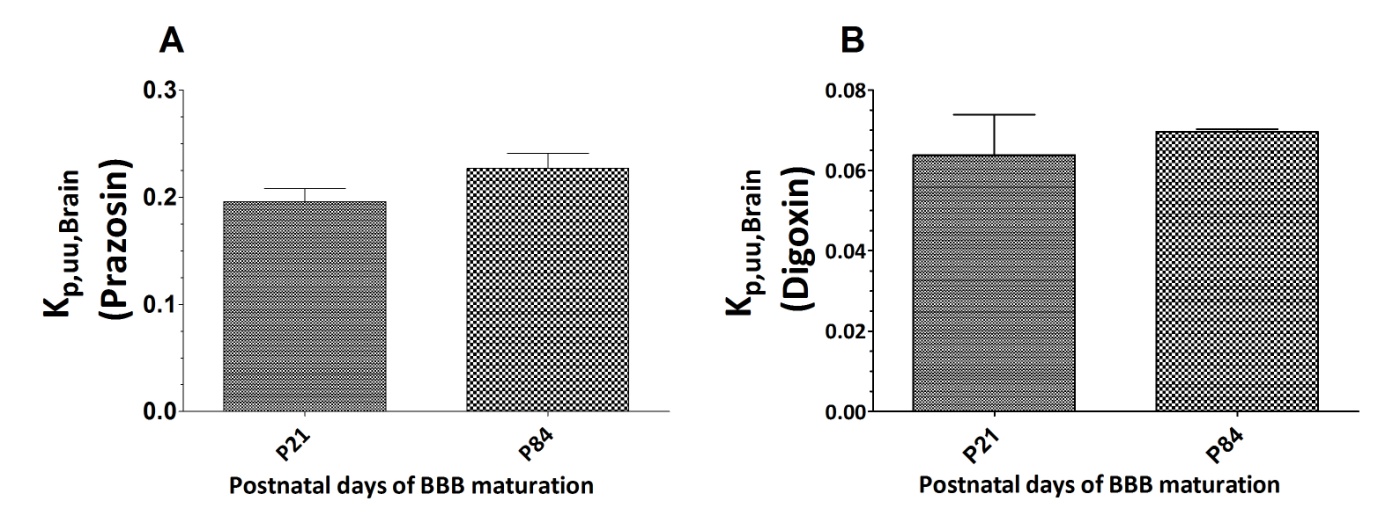
**

Supplement: Additional file 1 — Age-related changes in P-gp, and bcrp function at the BBB. Rats at pediatric (P21) and adult (P84) stages of braindevelopment were used. The in vivo plasma and brain exposure of tested efflux transporter substrates [digoxin (P-gp substrate): 0.5 mg/kg h,, and prasozin (bcrp substrate): 0.25 mg/kg h] was assessed using unanesthetized rats catheterized in the femoral vein. Drug infusion was performed for a period of 4 h (steady state), and then the unbound partitioning coefficient brain/plasma concentration ratio (Kpuu, brain) for each tested efflux transporter substrate was determined as described in the Experimental Section. Data represent the mean concentration for 3 or 5 rat brains and 3 or 5 plasmas; variability is given by bars ± SEM. Statistical comparisons: *P < 0.05, **P < 0.01, and ***P < 0.0001. [file 1742-2094-9-273-S1.doc]

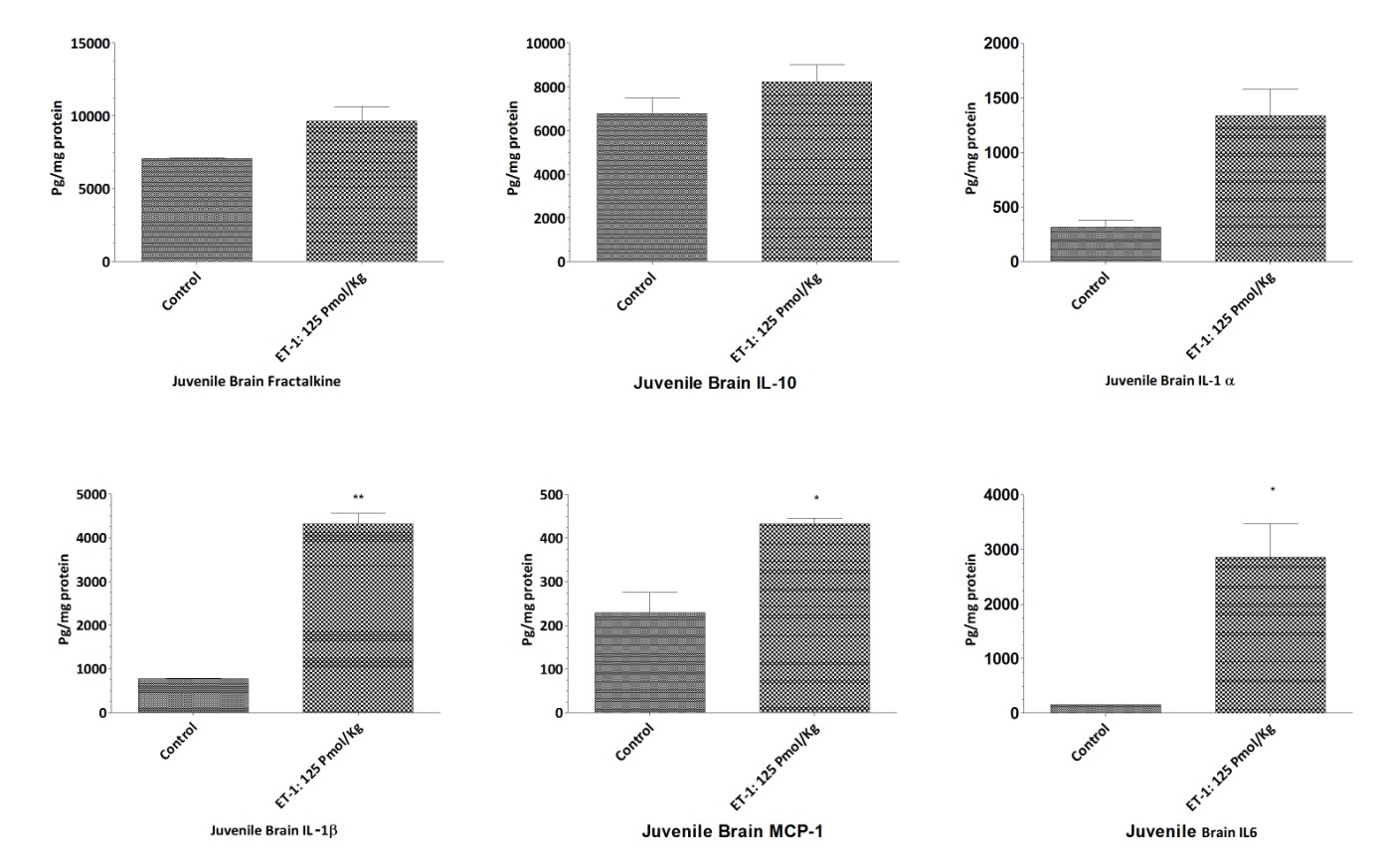

Supplement: Additional file 2 — Cytokines and chemokines levels in juvenile brain after a single intracerebral injection of endothelin-1 for 24 h at the dose of 125 pmol/kg. All cytokines, IL-1 β, IL-6, IL-10, MCP-1/ccl2, fractalkine, TIMP-1, IL-13, cinculin (CINC-1) were simultaneously measured in brain and serum samples using the Agilent technology cytokine microarray (Tebu bio). All brain values were corrected for individual serum measurements as follows and described previously [6]: Graph plotted as mean ± S.E.M. *P <.05, **P <0.01, ***P <0.001 compared with animal controls with n = 3 or 5 rats for each group. [file 1742-2094-9-273-S2.doc]
